# Ambulatory Smartwatch ECG Monitoring among Patients Undergoing Transcatheter Aortic Valve Replacement Early after Discharge: An Observational Study

**DOI:** 10.31083/j.rcm2401011

**Published:** 2023-01-04

**Authors:** Yi Zhang, Tian-Yuan Xiong, Xue-Mei Yang, De-Fang Chen, Yi-Ming Li, Yun Bao, Mao Chen

**Affiliations:** ^1^Department of Cardiology and Laboratory of Heart Valve Disease, West China Hospital, Sichuan University, 610041 Chengdu, Sichuan, China; ^2^Department of Nursing, West China Hospital, Sichuan University, 610041 Chengdu, Sichuan, China

**Keywords:** transcatheter aortic valve replacement, smartwatch, arrhythmia, ambulatory electrocardiogram monitor

## Abstract

**Background:**

As an emerging arrhythmia monitor, ambulatory smartwatch electrocardiogram (ECG) provides an option for home-based monitoring of delayed new-onset arrhythmic events after transcatheter aortic valve replacement (TAVR). We aimed to validate the diagnostic efficacy of a consumer smartwatch ECG in TAVR recipients, while further explore the occurrence rate of both tachy- and brady-arrhythmia for 30 days after discharge to support risk management.

**Methods:**

Consecutive TAVR recipients from February 26th, 2021 to December 13th, 2021 were enrolled prospectively, receiving simultaneous 24-hour Holter and 12-lead ECG compared with smartwatch ECG during hospitalization and daily smartwatch ECG collection for 30 days after discharge.

**Results:**

Among 110 patients, the efficacy of smartwatch ECG presented sensitivity and specificity in diagnosing atrial fibrillation (AF) as 1.00 and 0.97, left bundle branch block (LBBB) as 0.61 and 0.88, and right bundle branch block (RBBB) as 0.60 and 0.97, respectively, compared with 24-hour Holter; presented sensitivity and specificity in diagnosing AF as 0.88 and 1.00, LBBB as 0.90 and 0.96, and RBBB as 0.83 and 0.94, respectively, compared with 12-lead ECG. At 30-day follow-up, new-onset arrhythmia included new-onset severe conduction disturbance (SCD) (23.6%), new-onset AF (21.8%), new-onset permanent LBBB (14.5%) and new-onset permanent RBBB (0.9%); 69.2% (36/52) of early new-onset LBBB recovered at 30-day follow-up.

**Conclusions:**

The diagnostic efficacy of consumer smartwatch ECG in arrhythmic events among TAVR population was acceptable, which provided a recommendable option for home-based management.

**Clinical Trial Registration:**

*“Continuously ambulatory rhythm monitoring and predictors of electrocardio-related adverse events in 30 days after transcatheter aortic valve replacement”*; Identifier: ChiCTR2000041244; http://www.chictr.org.cn/showproj.aspx?proj=66324.

## 1. Introduction

Degenerative aortic stenosis accounts for more than 3% of people aged 75 years or older, with high mortality without proper intervention [1]. Transcatheter aortic valve replacement (TAVR) is now a guideline-recommended alternative therapy instead of surgery in terms of selected patients with severe aortic stenosis. The number of patients who received TAVR has reached more than 200 thousand in the United States [2]. However, postprocedural arrhythmic events including new-onset conduction disturbances and atrial fibrillation postprocedurally, are one of the most frequent complications of TAVR, which are also associated with worse clinical outcomes [3]. The new-onset conduction disturbance after TAVR is still one of those frequently encountered complications despite device iteration, still remaining the main drawback of the procedure. Atrial fibrillation (AF) is common among the TAVR population. Patients discharged with AF were associated with higher risk of mortality [4]. Moreover, the rates of new-onset high-degree atrial ventricular block (AVB) requiring permanent pacemaker implantation (PPI) and new-onset AF surge within the first month postprocedurally, and then decrease thereafter [5, 6]. The prevalence of new-onset severe bradyarrhythmia and new-onset AF can be approximately 9.8% and 81.5% respectively within 1 month after TAVR [7, 8]. However, data on daily arrhythmia monitoring early after TAVR are still insufficient.

Ambulatory electrocardiogram (ECG) has been used for post-TAVR monitoring primarily by real-time cardiovascular telemetry monitors or invasively implantable monitors to delineate arrhythmic events post-TAVR, but is limited by frequent electrode changes, cost or the invasive nature [7, 9]. As an emerging wearable device for health monitoring, consumer smartwatches have ECG monitoring functions with the advantages of portability, high acceptance and low cost. The efficacy of smartwatch ECG in screening and diagnosing AF has been preliminarily verified [10]. On account of the expansion of TAVR indications, the management of post-TAVR arrhythmic events is increasingly important. Therefore, more evidence is required to elucidate the arrhythmic events postprocedurally and to guide risk management. Thus, we conducted an observational study prospectively to explore the occurrence rate of both tachy- and brady-arrhythmic events in TAVR recipients for 30-day follow-up based on a consumer smartwatch, after validation by in-hospital 12-lead ECG and 24-hour Holter ECG.

## 2. Methods

### 2.1 Patient Recruitment and Research Flow

Consecutive patients undergoing TAVR in our center were prospectively enrolled from February 26th, 2021 to December 13th, 2021. This clinical trial was a single-center, prospective and single-arm observational clinical study approved by the Institutional Ethics Committee. The administration was approved by the Chinese Clinical Trial Registry Center (http://www.chictr.org.cn) on December 22nd, 2020 (registration number: ChiCTR2000041244).

Inclusion criteria included (i) patients with TAVR indication after the heart team’s discretion; (ii) patients having smartphones based on the Android or IOS system with the ability to download and use the “Midong health” application; and (iii) patients able to use the smartwatch independently after teaching. Exclusion criteria included: (i) patients refusing to wear the smartwatch; (ii) patients unable to use the smartwatch due to impaired cognitive function, bilateral upper extremity disability or allergic reaction to smartwatch material; and (iii) patients unable to acquire accurate smartwatch data due to abnormal skin color, severe occlusive vascular disease or obvious edema of the upper extremity.

**Fig. 1.**
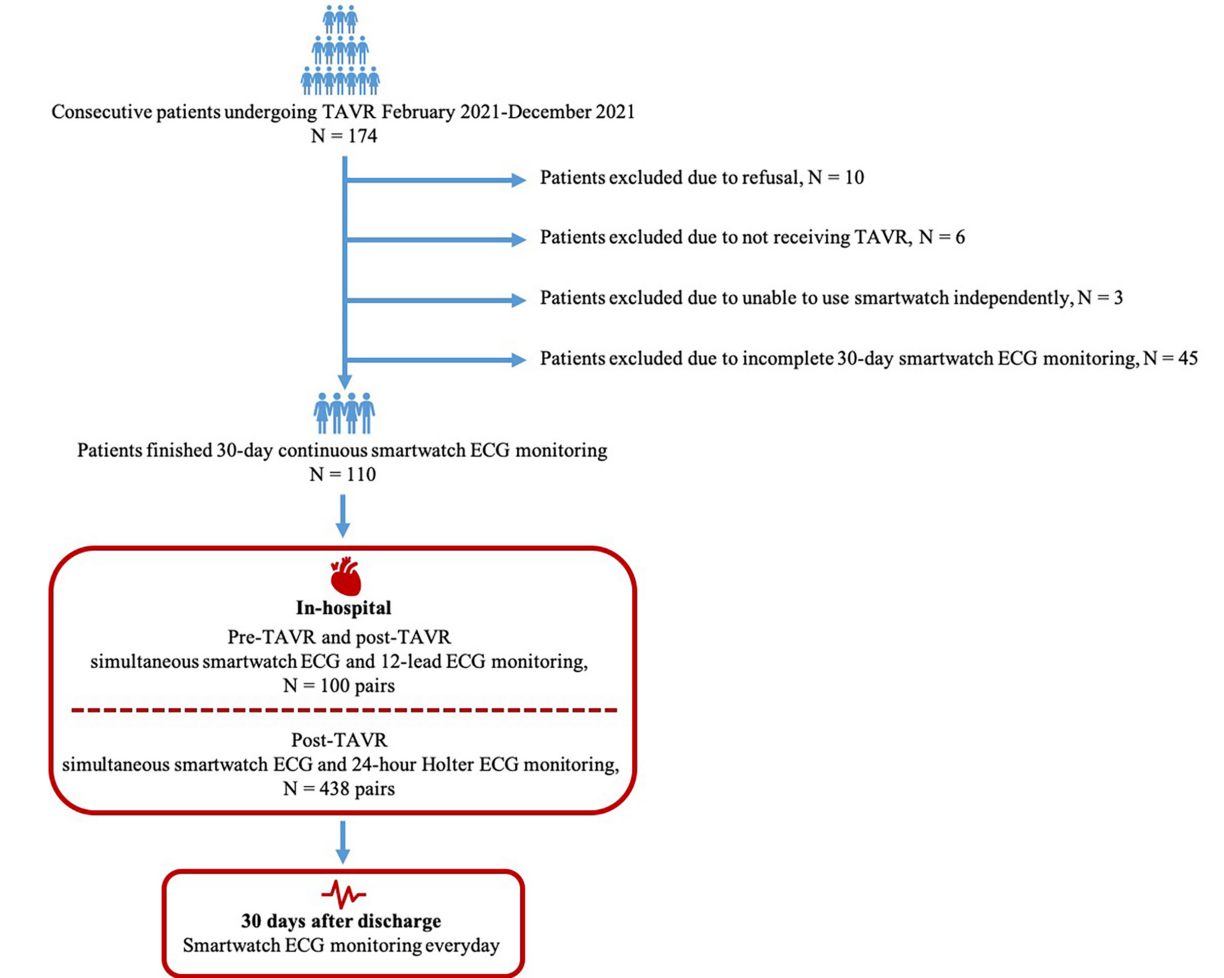
**Flowchart of main study process**. TAVR, transcatheter aortic valve replacement; N, number; ECG, electrocardiograph.

The research process was shown in Fig. 1. After comprehensively preoperative evaluation, patients who met the inclusion criteria underwent informed consent. After that, enrolled patients were taught the detailed process to collect smartwatch ECG by inpatient nurses. The single-lead smartwatch ECG was actively collected by patients. During ECG collection, the back electrode of the smartwatch clung to the skin of the unilateral wrist, and the fingers of the contralateral upper extremity pressed on the front electrode of the smartwatch. Each ECG collection duration time was 60 seconds. After patients mastered the ECG collection process, the simultaneous acquisition of bedside 12-lead ECG and smartwatch ECG was conducted at least twice a day preoperatively, with patients in the supine position. The smartwatch ECG would be uploaded to the online Midong Health Physician’s Management Platform via a smartphone which connected to the smartwatch through Bluetooth. Investigators needed to confirm the completeness and accuracy of the online data. Patients were routinely monitored by 24-hour Holter ECG after TAVR combined with smartwatch ECG during the same period. After the completion of the 24-hour Holter ECG acquisition, the simultaneous collection of bedside 12-lead ECG and smartwatch ECG was resumed until discharge. After discharge, patients wore the smartwatch continuously for 30 days, collecting smartwatch ECG every morning, afternoon and night, except when smartwatch was charging. The simultaneous 24-hour Holter ECG and 12-lead ECG were not collected after discharge. Moreover, initiating the “Midong Health” application on the smartphone should be achieved at least once a day to successfully upload data to the online platform. Additional smartwatch ECG collection should be conducted if patients had obvious symptoms such as dizziness, palpitations, amaurosis or syncope, etc. Physicians needed to evaluate all smartwatch ECG after discharge on the online Midong Health Physician’s Management Platform every day and confirmed and replied to patients’ message. Major or urgent adverse arrhythmic events included new-onset AF, ventricular fibrillation, ventricular tachycardia with heart rate >150 beats/minute, second- or third-degree AVB or other high-degree AVB, sustained bradycardia with heart rate <30 beats/minute for more than 30 seconds and asystole with consciousness for more than 2 seconds. Investigators were required to conduct urgent telecommunication for major or urgent arrhythmic events. Other tachycardic or bradycardic events that did not meet the aforementioned standard were inquired by text or voice communication through the application. Inquiries were also sent if there were no smartwatch data received. Anticoagulant therapy should be initiated in patients with new-onset AF episodes lasting >5.5 hours daily or >6 minutes daily along with ${\mathrm{CHA}}_{2}$${\mathrm{DS}}_{2}$VASc scores ≥3 after discharge. Patients needed to return the smartwatch and finish echocardiographic assessment 30 days after discharge.

### 2.2 Smartwatch and Data Processing

The smartwatch used in this study was reported previously (configuration in **Supplementary Fig. 1**) [11, 12, 13, 14]. The biomonitoring function of the smartwatch named Amazfit used in this study had the same core structure and data process algorithm as a former version from the same company (Anhui Huami Information Technology Co. Ltd.), which has been approved for medical use by the National Medical Products Administration in China (Anhui Device Registration Approval No. 20182210012). This study mainly involved the function of this smartwatch in collecting single-lead ECG. The collecting process was activated by the patients themselves, with a sampling frequency of 250 Hz. Each ECG collection duration was 60 seconds, forming a single-lead ECG similar to the limb I ECG of the traditional 12-lead ECG. A high-precision optical sensor, the photoplethysmograph (PPG), automatically collected the pulse signal on the back of the smartwatch at a frequency of 50 Hz. The data collected by the smartwatch were transmitted to the “Midong health” application on a smartphone through Bluetooth, and further transmitted to the online Midong Health Physicians’ Management Platform through the Internet. The data were further analyzed by an artificial intelligence engine (RealBeats Artificial Intelligence Biological Data Engine), which was trained and verified by deep a convolutional neural network (SEResNet). The smartwatch, “Midong health” application and online Midong Health Physicians’ Management Platform were all provided by the same company.

### 2.3 PPI Criteria

In our center, PPI would be implanted with informed content and at least one of these criteria: (i) high-degree atrial ventricular block (AVB) (second-degree Mobitz II, third-degree AVB or other high-degree AVB); (ii) sick sinus syndrome with symptoms; (iii) first-degree AVB and persistent left bundle branch block; (iv) severe bradyarrhythmia dependent on temporary cardiac pacing.

### 2.4 Outcomes of Interest and Definitions

The arrhythmic events of interest included early new-onset AF, early new-onset left bundle branch block (LBBB), early new-onset right bundle branch block (RBBB), early new-onset severe conduction disturbance (SCD); delayed new-onset AF, delayed new-onset LBBB, delayed new-onset RBBB, delayed new-onset SCD; new-onset AF, new-onset permanent LBBB, new-onset permanent RBBB, new-onset SCD. Early new-onset AF, LBBB, and RBBB were defined as corresponding new-onset arrhythmia postoperatively detected by 12-lead ECG, 24-hour Holter ECG or smartwatch ECG before discharge. Early new-onset SCD was defined as new-onset SCD (including third-degree or other high-degree AVB, sustained pacing rhythm or RR interval greater than 2 seconds on smartwatch ECG) postoperatively detected by 12-lead ECG, 24-hour Holter ECG or smartwatch ECG before discharge. If the diagnostic results of smartwatch ECG were inconsistent with the in-hospital 24-hour Holter ECG or 12-lead ECG, the latter two were regarded as the gold standard. Delayed new-onset AF, LBBB, and RBBB were defined as new-onset corresponding arrhythmias detected by smartwatch ECG within 30 days after discharge. Delayed new-onset SCD was defined as new-onset SCD (including third-degree or other high-degree AVB or RR interval greater than 2 s) detected by smartwatch ECG within 30 days after discharge. New-onset permanent LBBB and RBBB were defined as corresponding early new-onset LBBB and RBBB presented persistently on the smartwatch ECGs every day for 30 days after discharge. New-onset AF or SCD meant early new-onset AF or SCD plus delayed new-onset AF or SCD. The diagnosis of AF, LBBB, RBBB, and SCD of the smartwatch ECG was based on the interpretation of two cardiologists (Y. Z. and TY. X.), without awareness of the corresponding 12-lead ECGs or 24-hour Holter ECGs. Disagreements were settled by consensus. The diagnosis of AF, LBBB, RBBB, and SCD on 24-hour Holter ECGs was conducted by electrocardiographic specialists in the Department of Electrocardiogram, without the awareness of the corresponding smartwatch ECG results. The diagnosis of AF, LBBB, RBBB, and SCD 12-lead ECGs was conducted by physicians in the Department of Cardiology, without the awareness of the corresponding smartwatch ECG results.

The diagnostic criteria for RBBB on smartwatch ECG were (i) QRS duration ≥120 milliseconds; and (ii) S wave duration was longer than R wave or >40 milliseconds. The diagnostic criteria for LBBB on smartwatch ECG were (i) QRS duration ≥120 milliseconds and (ii) blunt or wide notch on R wave. The diagnostic criteria for AF on smartwatch ECG were (i) irregular R-R intervals; (ii) absence of distinct repeating P waves; and (iii) irregular atrial activations. The diagnostic criteria for SCD on smartwatch ECG were at least one of the following conditions: (i) second-degree type 2 AVB when QRS ≥120 milliseconds; (ii) AVB with 2:1 conduction when QRS ≥120 milliseconds; (iii) at least 2 consecutive sinus P waves at constant physiologic frequency did not conduct to ventricle, or the presence of RR intervals greater than 2 s; (iv) prolonged asystole time (>3 s) or sustained ventricular bradyarrhythmia (<50 beats/min) based on AF rhythm; (v) constant P wave in stable frequency with isolated ventricular rhythm (no correlation between P and R waves), or a fixed slow ventricular rhythm in the presence of AF. The clinical outcomes of interest mainly included rehospitalization and mortality at 30-day follow-up.

### 2.5 Statistical Analysis

Continuous variables with normal distributions were represented as the mean value ± standard deviation. Continuous variables with skewed distributions were represented as the median value [25th percentile, 75th percentile]. Categorical variables were represented by frequency (%). Logistic regression was used for the analysis of risk predictors of new-onset arrhythmic events. Factors with a *p* value < 0.1 in univariate regression analysis and with clinical significance were included in further multivariate regression analysis. The diagnostic performance of the smartwatch ECG was evaluated by sensitivity, specificity, positive predictive value, negative predictive value and their corresponding 95% confidence intervals (CI) [15, 16]. In addition, the minimalist sample size of this diagnostic study was calculated based on a previous study using smartwatch from the same company, with predicted sensitivity as 0.87, predicted specificity as 0.99, permitted error rate as 0.025, two-sided error rate α as 0.05, and withdraw rate as 20% [11, 17, 18, 19, 20]. All analyses involved in this study were based on SPSS software (version 26.0.0.0, IBM Corp., Chicago, IL, USA) and R software (version 4.1.0, R Foundation for Statistical Computing, Vienna, Autria). A *p* value < 0.05 was considered to be statistically significant.

## 3. Results

### 3.1 Baseline Information and Post-TAVR In-Hospital Outcomes

Consecutive TAVR patients in our center from February 26th, 2021 to December 13th, 2021 were enrolled, with final analysis of 110 patients who completed daily smartwatch monitoring for 30 days after discharge (Fig. 1). The evaluated minimalist sample size was 55 patients. The median age of the included patients was 72 years. The median STS-PROM score was 2.50%. For the preoperatively arrhythmic events, AF, LBBB, RBBB and AVB accounted for 21.8% (24/110), 4.5% (5/110), 4.5% (5/110), and 17.4% (19/110), respectively (Table 1). During the TAVR operation, patients who received self-expanding prostheses and first-generation prostheses reached 92.7% (101/110) and 55.5% (61/110), respectively. After TAVR, the proportion of patients who received PPIs reached 16.4% (18/110) (Table 1).

**Table 1.** **Baseline information of patients with accomplished 30-day follow-up**.

Parameter		All (N =110)
Demography and medical history		
	Age, year	72.0 [67.0, 76.0]
	BMI, ${\mathrm{kg\cdot m}}^{–\,2}$	22.7 [19.8, 24.5]
	Male (%)	62 (56.4%)
	STS-PROM, %	2.5 [1.6, 3.3]
	NYHA III-IV (%)	81 (73.6%)
	CAD (%)	69 (62.7%)
	Renal dysfunction (%)	8 (7.3%)
	Pre-TAVR statins (%)	47 (42.7%)
Pre-TAVR arrhythmic events		
	PPI (%)	3 (2.7%)
	AF (%)	24 (21.8%)
	LBBB (%)	5 (4.5%)
	RBBB (%)	5 (4.5%)
	AVB (%)	19 (17.4%)
Pre-TAVR echocardiography and CT assessment		
	${\mathrm{PG}}_{\text{mean}}$, mmHg	52.0 [42.0, 72.3]
	$V_{\text{max}}$, ${\mathrm{m\cdot s}}^{–\,1}$	4.82 ± 0.77
	LVEF, %	60.5 [51.3, 68.3]
	Bicuspid aortic valve (%)	67 (60.9%)
	Severe aortic stenosis (%)	78 (70.9%)

BMI, body mass index; STS-PROM, Society of Thoracic Surgeons predicted rate of mortality; NYHA, New York Heart Association; CAD, coronary artery disease; TAVR, transcatheter aortic valve replacement; AF, atrial fibrillation; LBBB, left bundle branch block; RBBB, right bundle branch block; AVB, atrial ventricular block; ${\mathrm{PG}}_{\text{mean}}$, mean value of transaortic valve pressure gradient; $V_{\text{max}}$, maximum velocity of transaortic valve blood flow; LVEF, left ventricular ejection fraction.

After TAVR, AF, AVB, LBBB, and RBBB accounted for 20.9% (23/110), 42.7% (47/110), 50.0% (55/110) and 10.0% (11/110), respectively. Early new-onset AF, early-onset new-onset LBBB, early-onset new-onset RBBB and early-onset new-onset SCD accounted for 7.3% (8/110), 47.3% (52/110), 6.4% (7/110) and 15.5% (18/110), respectively, with median occurrence times of 4.5 days, 4.0 days, 3.0 days and 2.0 days after TAVR, respectively (Table 2).

**Table 2.** **Post-TAVR in-hospital outcomes of patients with accomplished 30-day follow-up**.

Parameter		All (N =110)
Intra-TAVR information		
	Self-expanding prosthesis (%)	102 (92.7%)
	First generation prosthesis (%)	61 (55.5%)
	Pre-dilation (%)	93 (84.5%)
	Post-dilation (%)	54 (49.1%)
	THV implantation depth, mm	5.0 [2.7, 8.8]
Post-TAVR clinical outcomes and assessment		
	New PPI (%)	18 (16.4%)
	PPI time, ${\mathrm{day}}^{†}$	5 [1, 6]
	Post-TAVR hospitalization duration, day	6.0 [5.0, 8.0]
	$V_{\text{max}}$, ${\mathrm{m\cdot s}}^{–\,1}$	2.4 [2.0, 2.8]
	${\mathrm{PG}}_{\text{mean}}$, mmHg	13.0 [8.8, 18.0]
	LVEF, %	61.5 [47.5, 68.3]
Post-TAVR arrhythmic events		
	AF (%)	23 (20.9%)
	AVB (%)	47 (42.7%)
	LBBB (%)	55 (50.0%)
	RBBB (%)	11 (10.0%)
	Early new-onset AF (%)	8 (7.3%)
	Early new-onset AF time, ${\mathrm{day}}^{†\,†}$	4.0 [2.3, 5.0]
	Early new-onset LBBB (%)	52 (47.3%)
	Early new-onset LBBB time, ${\mathrm{day}}^{†\,†}$	4.0 [2.0, 6.0]
	Early new-onset RBBB (%)	7 (6.4%)
	Early new-onset RBBB time, ${\mathrm{day}}^{†\,†}$	2.0 [2.0, 10.0]
	Early new-onset SCD (%)	17 (15.5%)
	Early new-onset SCD time, ${\mathrm{day}}^{†\,†}$	3.0 [0, 4.0]

TAVR, transcatheter aortic valve replacement; PPI, permanent pacemaker implantation; THV, transcatheter heart valve; $V_{\text{max}}$, maximum velocity of transaortic valve blood flow; ${\mathrm{PG}}_{\text{mean}}$, mean value of transaortic valve pressure gradient; LVEF, left ventricular ejection fraction; AF, atrial fibrillation; LBBB, left bundle branch block; RBBB, right bundle branch block; SCD, severe conduction disturbances. ^†^PPI time meant time between TAVR and PPI procedure. ^††^Early new-onset arrhythmia time meant time between TAVR procedure and first day of new-onset arrhythmia.

### 3.2 Diagnostic Efficacy of Smartwatch ECG

The diagnostic efficacy of smartwatch ECG in terms of AF, LBBB, and RBBB was validated by 100 paired simultaneous 24-hour Holter ECGs and 438 paired 12-lead ECGs (**Supplementary Tables 1,2**). When compared with 24-hour Holter ECG, the sensitivity, specificity, positive predictive value (PPV), and negative predictive value (NPV) of smartwatch ECG in the diagnosis of AF were 1.00 (95% CI 0.66–1.00), 0.97 (95% CI 0.89–0.99), 0.77 (95% CI 0.46–0.94), and 1.00 (95% CI 0.94–1.00), respectively; the sensitivity, specificity, PPV, and NPV of smartwatch ECG in the diagnosis of LBBB were 0.61 (95% CI 0.41–0.78), 0.88 (95% CI 0.78–0.94), 0.68 (95% CI 0.46–0.84), and 0.84 (95% CI 0.74–0.92), respectively; the sensitivity, specificity, PPV, and NPV of smartwatch ECGs in the diagnosis of RBBB were 0.60 (95% CI 0.17–0.93), 0.97 (95% CI 0.90–0.99), 0.50 (95% CI 0.14–0.86), and 0.97 (95% CI 0.91–1.00), respectively (Table 3).

**Table 3.** **Diagnostic efficacy of smartwatch ECG for AF, LBBB and RBBB evaluated by sensitivity, specificity, PPV and NPV**.

		AF	LBBB	RBBB
		estimate [95% CI]	estimate [95% CI]	estimate [95% CI]
24-hour Holter ECG				
	Sensitivity	1.00 [0.66–1.00]	0.61 [0.41–0.78]	0.60 [0.17–0.93]
	Specificity	0.97 [0.89–0.99]	0.88 [0.78–0.94]	0.97 [0.90–0.99]
	PPV	0.77 [0.46–0.94]	0.68 [0.46–0.84]	0.50 [0.14–0.86]
	NPV	1.00 [0.94–1.00]	0.84 [0.74–0.92]	0.97 [0.91–1.00]
12-lead ECG				
	Sensitivity	0.88 [0.76–0.95]	0.90 [0.82–0.94]	0.83 [0.60–0.94]
	Specificity	1.00 [0.98–1.00]	0.96 [0.94–0.98]	0.94 [0.91–0.96]
	PPV	0.98 [0.87–1.00]	0.90 [0.82–0.94]	0.45 [0.30–0.61]
	NPV	0.98 [0.96–0.99]	0.96 [0.94–0.98]	0.99 [0.97–1.00]

ECG, electrocardiograph; AF, atrial fibrillation; LBBB, left bundle branch block; RBBB, right bundle branch block; PPV, positive predictive value; NPV, negative predictive value.

Taking simultaneous 12-lead ECG as the gold standard, the sensitivity, specificity, PPV, and NPV of smartwatch ECG in the diagnosis of AF were 0.88 (95% CI 0.76–0.95), 1.00 (95% CI 0.98–1.00), 0.98 (95% CI 0.87–1.00), and 0.98 (95% CI 0.96–0.99), respectively; the sensitivity, specificity, PPV, and NPV of smartwatch ECG in the diagnosis of LBBB were 0.90 (95% CI 0.82–0.94), 0.96 (95% CI 0.94–0.98), 0.90 (95% CI 0.82–0.94), and 0.96 (95% CI 0.94–0.98), respectively; the sensitivity, specificity, PPV and NPV of smartwatch ECG in the diagnosis of RBBB were 0.83 (95% CI 0.60–0.98), 0.94 (95% CI 0.91–0.96), 0.45 (95% CI 0.30–0.61), and 0.99 (95% CI 0.97–1.00), respectively (Table 3).

### 3.3 Arrhythmic Events and Clinical Outcomes at 30-Day Follow-Up

At 30-day follow-up, the incidence of AF, LBBB, RBBB, and SCD diagnosed by smartwatch ECG was 39.1% (43/110), 44.5% (49/110), 7.3% (8/110), and 8.2% (9/110), respectively. Among the 9 patients with SCD, 33.3% (3/9) presented bradyarrhythmia-related symptoms. The incidence of delayed new-onset AF, delayed new-onset LBBB, and delayed new-onset SCD after discharge was 14.5% (16/110), 1.8% (2/110), and 7.3% (8/110), respectively. Patients with delayed new-onset AF after discharge had a median ${\mathrm{CHA}}_{2}$${\mathrm{DS}}_{2}$VASc score of 2 and a median AF episode duration of 1.2 minutes. The median occurrence times of delayed new-onset AF, delayed new-onset LBBB, and delayed new-onset SCD were 10.0 days, 6.0 days, and 15.0 days after TAVR, respectively (Table 4).

**Table 4.** **Arrhythmic events at 30-day follow-up of patients with accomplished 30-day follow-up**.

Arrhythmic events at 30-day follow-up		All (N = 110)
AF (%)		43 (39.1%)
LBBB (%)		49 (44.5%)
RBBB (%)		8 (7.3%)
SCD (%)		9 (8.2%)
Delay new-onset arrhythmic events		
	Delay new-onset AF (%)	16 (14.5%)
	Delay new-onset AF time, ${\mathrm{day}}^{†}$	10.0 [7.0, 15.3]
	${\mathrm{CHA}}_{2}$${\mathrm{DS}}_{2}$VASc scores of delay new-onset AF, point	2.0 [2.0, 3.8]
	Duration of delay new-onset AF, minute	1.2 [1.0, 1.7]
	Delay new-onset LBBB (%)	2 (1.8%)
	Delay new-onset LBBB time, ${\mathrm{day}}^{†}$	6.0 [4.5, 6.8]
	Delay new-onset SCD (%)	8 (7.3%)
	Delay new-onset SCD time, ${\mathrm{day}}^{†}$	15.0 [8.5, 21.5]
Overall new-onset arrhythmic events		
	New-onset AF (%)	24 (21.8%)
	New-onset AF time, ${\mathrm{day}}^{†\,†}$	7.0 [5.0, 12.0]
	New-onset permanent LBBB (%)	16 (14.5%)
	New-onset permanent LBBB time, ${\mathrm{day}}^{†\,†}$	3.0 [2.0, 6.0]
	New-onset permanent RBBB (%)	1 (0.9%)
	New-onset permanent RBBB time, ${\mathrm{day}}^{†\,†}$	2.0
	New-onset SCD (%)	26 (23.6%)
	New-onset SCD time, ${\mathrm{day}}^{†\,†}$	4.0 [0, 7.3]

AF, atrial fibrillation; LBBB, left bundle branch block; RBBB, right bundle branch block; SCD, severe conduction disturbances. ^†^Delayed new-onset arrhythmia time was time between TAVR and first day of delayed new-onset arrhythmia. ^††^New-onset arrhythmia time was time between TAVR and first day of new-onset arrhythmia.

**Fig. 2.**
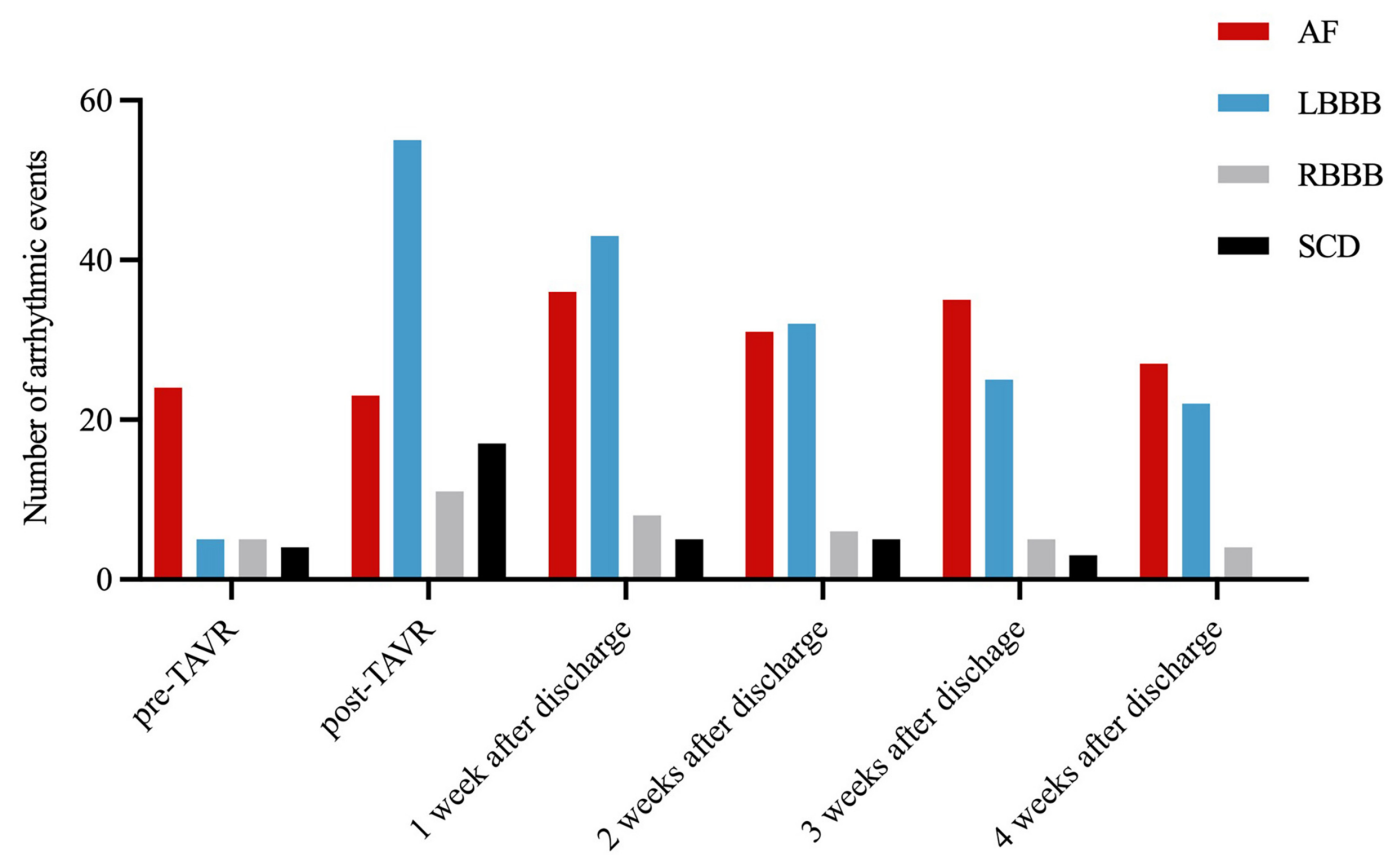
** The total number of AF, LBBB, RBBB and SCD observed pre-operatively, post-operatively, 1 week after discharge, 2 weeks after discharge, 3 weeks after discharge and 4 weeks after discharge**. TAVR, transcatheter aortic valve replacement; AF, atrial fibrillation; LBBB, left bundle branch block; RBBB, right bundle branch block; SCD, severe conduction disturbance.

In total, the incidence rates of overall new-onset AF, new-onset permanent LBBB, new-onset permanent RBBB, and new-onset SCD after TAVR were 21.8% (24/110), 14.5% (16/110), 0.9% (1/110), and 23.6% (26/110), respectively. A total of 69.2% (36/52) of early new-onset LBBB recovered at the 30-day follow-up. The median occurrence times of new-onset AF, new-onset permanent LBBB, new-onset permanent RBBB, and new-onset SCD were 7.0 days, 3.0 days, 2.0 days, and 4.0 days after TAVR, respectively (Table 4). The total numbers of AF, LBBB, RBBB and SCD observed preoperatively, postoperatively, 1week after discharge, 2 weeks after discharge, 3 weeks after discharge and 4 weeks after discharge were displayed in Fig. 2.

There were 110 patients completing the 30-day follow-up (Table 5). At 30-day follow-up, the rate of rehospitalization was 1.8% (2/110), and the rate of death was 0. Moreover, the mean values of $V_{\text{max}}$ and ${\mathrm{PG}}_{\text{mean}}$ were 2.4 m⋅$s^{-1}$ and 12 mmHg, respectively. The rate of moderate aortic regurgitation reached 1.8% (2/110). The value of left ventricular ejection fraction assessed by echocardiography was 61% on average.

**Table 5.** ** Clinical outcomes including echocardiographic assessment of included patients at 30-day follow-up**.

Parameter	All (N = 110)
$V_{\text{max}}$, ${\mathrm{m\cdot s}}^{–\,1}$	2.4 [2.0, 2.7]
${\mathrm{PG}}_{\text{max}}$, mmHg	12.0 [9.0, 17.0]
moderate AR (%)	2 (1.8%)
LVEF, %	61.0 [53.0, 68.0]
Re-hospitalization (%)	2 (1.8%)
Re-hospitalization time, day	15.0 ± 2.8
Death (%)	0

$V_{\text{max}}$, maximum velocity of transaortic valve blood flow; ${\mathrm{PG}}_{\text{mean}}$, mean value of transaortic valve pressure gradient; AR, aortic regurgitation; LVEF, left ventricular ejection fraction.

## 4. Discussion

We analyzed post-TAVR arrhythmic events until 30 days after discharge mainly detected by smartwatch ECG in our single-center study. Our results demonstrated that smartwatch ECG showed acceptable diagnostic performance when compared with 24-hour Holter ECG and 12-lead ECG in diagnosing AF, LBBB and RBBB. At 30-day follow-up, the overall new-onset arrhythmic events were new-onset SCD, new-onset AF, new-onset permanent LBBB, and new-onset permanent RBBB in the order of most to least proportion. Over half of the early new-onset LBBB recovered within 30 days after discharge.

### 4.1 New-Onset Conduction Disturbances Early after TAVR

Patients with new-onset SCD or new-onset permanent LBBB accounted for the majority of arrhythmic subjects at 30-day follow-up after TAVR. Patients who received PPI accounted for 16.4% (18/110), with the indication of new-onset SCD reaching 88.9% (16/18). All aforementioned PPIs were conducted during index hospitalization. A recent systematic review presented that the overall rate of PPI after TAVR with early- and new-generation valves ranged from 2.3% to 37.7% [21]. A previous study including subjects receiving mobile cardiac telemetry monitoring after TAVR presented 9% (21/245) of high-degree AVB or complete heart block leading to PPI at 30-day follow-up, of which the majority (75%) were asymptomatic [3]. For most patients with delayed new-onset SCD after discharge in our study, the absence of bradyarrhythmia-related symptoms was also observed. This might be related to a relatively low degree of atrioventricular block (AVB), short duration time of brady-arrhythmic attack and inappropriate judgment of smartwatch ECG due to pitfall caused by single-lead ECG. Previous studies also reported a major proportion of transient and asymptomatic delayed new-onset high-degree AVB [9, 22]. In our study, a total of 3 patients presented with SCD-related symptoms whose ECGs were captured by smartwatch during follow-up. The symptoms disappeared, and ECGs recovered after out of touch with the electrical cord (1 patient with PPI) or suspension of beta-blocker (2 patients without PPI). We did not find any delayed new-onset SCD that needed PPI at 30-day follow-up. This might be related to the relatively longer hospitalization duration (6.8 days on average) of our included patients compared with the next-day discharge achieved mostly in other studies [23, 24]. Thus, a longer in-hospital monitoring time might result in the timely detection and treatment of new-onset SCD that requires PPI during hospitalization, with a median time of 5 days (interquartile range: 1 to 6) from TAVR to PPI, which was similar to other investigations [25]. We also analyzed risk predictors for new-onset arrhythmias additionally (**Supplementary Table 3**). Our results suggested that baseline RBBB and statin usage were independent predictors of new-onset SCD. Consistently, a review has recommended 2 to 4 weeks of ambulatory ECG monitoring after TAVR in all patients with baseline RBBB discharged without PPI [3]. No related studies have indicated an association between statins and conduction disturbances in the TAVR population. Complete AVB might be caused by hyperkalemia due to rhabdomyolysis after using statins [26].

Rates of new-onset LBBB after TAVR ranged from 4% to 65% depending on the type of prosthesis, presented 9% to 65% in subjects receiving CoreValve and 4% to 18% in subjects receiving Edwards Sapien valve [27]. Apart from the above, the majority of early new-onset LBBB seemed to decrease over time after discharge, which might be related to the remission of edema and/or inflammation caused during the TAVR procedure in the adjacent area of the cardiac conduction system. As progressive first AVB with LBBB and worsening or new onset LBBB were commonly reported indications of PPI, the aforementioned conditions make the decision to PPI difficult. Previous studies reported many predictors of new-onset LBBB such as female sex, first-degree AVB, and lower implantation depth [28]. After excluding spontaneously resolved early new-onset LBBB, we did not find any independent predictors for new-onset permanent LBBB (**Supplementary Table 3**).

### 4.2 New-Onset Atrial Fibrillation Early After TAVR

Patients presented new-onset AF reaching from 8.6% to 16.9% in studies at different risk profiles at 30-day follow-up [29, 30, 31]. The first report of subjects receiving implantable cardiac monitors after TAVR presented at least 73.35% of new-onset AF within the first month after the procedure [3]. Our results indicated that new-onset AF after TAVR was not rare at 30-day follow-up, which was in concordant with a previous study [3]. This implied the effectiveness and necessity of home-based AF monitoring to guide changes in anticoagulation therapy. In one previous clinical trial including TAVR recipients with new-onset persistent LBBB, nearly 8.3% of the enrolled population who presented new-onset AF received newly started anticoagulation treatment [3]. Research recommended initiating anticoagulation therapy in patients who presented with a daily AF duration >5.5 hours and/or a daily AF duration >6 minutes combined with a ${\mathrm{CHA}}_{2}$${\mathrm{DS}}_{2}$VASc score ≥3 [3]. However, patients with new-onset AF in our study presented a short AF episode duration (1.2 minutes on average) and low risk of stroke (2 of ${\mathrm{CHA}}_{2}$${\mathrm{DS}}_{2}$VASc score on average). Therefore, no patient received newly initiated anticoagulation therapy. This also indicated the high sensitivity of smartwatch ECG in detecting AF. As a relatively elderly and commonly antiplatelet-undertaking population, TAVR recipients should be given individualized consideration for anticoagulation therapy after balancing stroke and bleeding in the setting of AF. Previous studies have reported that nontransfemoral access (the strongest predictor), age, worse functional status, etc., were independent risk factors for new-onset AF after TAVR [32]. However, we did not find any significant predictors in our study, nor did we include patients who received TAVR through nontransfemoral access (**Supplementary Table 3**). Moreover, we did not find a significant difference in 30-day clinical outcomes (stroke and rehospitalization) between patients with and without new-onset AF due to the small sample size of our study.

### 4.3 Efficacy of Smartwatch ECG in Diagnosing Arrhythmias among the TAVR Population

To our knowledge, this was the first study to validate of the efficacy of consumer smartwatch ECG in diagnosing AF, LBBB and RBBB in a TAVR population. Our results suggested that the consumer smartwatch ECG demonstrated acceptable efficacy in diagnosing AF, LBBB, and RBBB. However, the sensitivity of smartwatch ECG in diagnosing LBBB and RBBB was poorer than that of 24-hour Holter ECG and with 12-lead ECG. This might be related to the presence of transient LBBB or RBBB detected by real-time 24-hour Holter monitors. Previous studies have demonstrated that the performance of smartwatch ECG from different companies in diagnosing AF was satisfactory [11, 33, 34]. The accuracy of AF diagnosis based on single-lead ECG with artificial intelligence algorithm of the smartwatch used in our study was identified before as well, demonstrating promising results (sensitivities: 88.68% and 96.67%; specificities: 100% and 98.01%) [11, 13]. Apart from smartwatch ECG, the screening of AF is usually recommended by passive detection through photoplethysmography coupled with artificial intelligence algorithms [35]. However, we judged all smartwatch ECGs artificially by investigators because of a lack of algorithms for diagnosing LBBB and RBBB. There have been no studies about validating of efficacy of smartwatch ECG in diagnosing SCD, either in our results. The ECG of SCD early after TAVR, which was anticipated to be detected by 24-hour Holter and smartwatch was difficult to collect simultaneously. In our study, one patient presenting with high-degree AVB after TAVR, which was detected by both smartwatch ECG and 12-lead ECG, received PPI 6 days after TAVR during index hospitalization. Moreover, a total of 9 patients had SCD events detected at home at 30-day follow-up. Three of the aforementioned patients presented symptoms related to bradyarrhythmia, of which 2 received concordant changes in medical treatment. This indicated the value of smartwatch ECG when combined with physicians’ discretion for monitoring SCD in post-TAVR recipients at home.

The rates of new-onset arrhythmias after TAVR by daily ECG monitoring in our study or the aforementioned similar studies for continuous ECG monitoring seemed to be relatively high. The reasons might be as follows: (i) Continuous ECG monitoring is more sensitive to those transient or asymptomatic new-onset arrythmias than traditional 12-lead ECG because of the longer duration or higher frequency of ECG monitoring. The high proportions of transient or asymptomatic new-onset arrhythmias after TAVR detected by continuously ambulatory monitors were also commonly reported [3]. (ii) Most patients in our center received the first-generation bioprosthesis (55.5%) and self-expanding bioprosthesis (92.7%), which were prone to have a higher risk of new-onset conduction disturbances after TAVR than the new-generation bioprosthesis and balloon expandable bioprosthesis.

### 4.4 Smartwatch for Remote Health Care in the TAVR Population

A previous study has proposed that smartwatch is valuable in clinical trials, as a tool for assessing patient-reported outcomes via wireless telecommunication, which we also used to guide remote health care (drug withdrawal, symptoms inquiry, health advice, etc.) [36]. Smartwatches with similar functions of single-lead ECG collection have also been reported in other brands, such as Apple Watch and Huawei Smartwatch [10, 23]. The telecare function of smartwatch might be especially demanded during the COVID-19 pandemic nowadays to overcome the difficulty in achieving in-site outpatient assessment. The smartwatch in our trial had telecare monitoring of sleep and activity. We also analyzed the daily sleep, step and heartbeat of our population, but no difference was found between 30 days after discharge and periprocedural time (**Supplementary Fig. 2**). The incomplete 30-day ECG monitoring data of some patients (29%, 45/155) in our study revealed a certain practical threshold of smartwatch for elderly patients, especially in terms of the difficulty of digital devices combined with Internet application. However, with the assistance from family members and telecommunication guidance from doctors, the compliance of patients in our study was satisfactory by and large. The smartwatch we used in this study was sold to approximately 97 U.S. dollars in China. The smartwatch was a cost-saving device for arrhythmia monitoring when compared with other cardiac telemetry monitors such as implantable cardiac monitors, and with the expense of the TAVR operation. Wearable devices with biomonitoring functions will be increasingly used due to the low cost. Moreover, their advantage in terms of the extreme clinical and practical usefulness to observe changes in health state and anomaly documents was also predictive of potential serious and disabling complications. Different types of smartwatches are commonly used by many other people in society for health monitoring. This could also avoid reminding patients as being ill and affecting their social life. Moreover, it provided an option to meet early-discharge demands nowadays while lowering the risk of major or urgent health events at home, achieving timely contact with patients and guiding changes in medical management.

### 4.5 Limitations

Our study had some limitations: (i) Due to the interfering vulnerability of single-lead smartwatch ECG, the accuracy of diagnosis of smartwatch ECG might be affected because of the poor image quality. However, after the validation of in-hospital 12-lead ECG and 24-hour ECG, the diagnostic performance of smartwatch ECG was acceptable. (ii) The included sample size of patients was small due to single-center enrollment, which also impeded further grouping or comparative analysis. However, this study might open the prospect of the possibility of icloud-based monitoring by a low-cost wearable device for the main multinationals of TAVR devices to investigate arrhythmias among recipients. (iii) The training of older patients to use smartwatches was more complicated to apply, as the average age of patients in our study was 72 years. However, it could be very useful to use the smartwatch in low-risk patients who are usually younger and more familiar with digital technologies.

## 5. Conclusions

The diagnostic efficacy of consumer smartwatch ECG in diagnosing LBBB, RBBB and AF among the TAVR population was acceptable. After TAVR, new-onset SCD and AF were the most frequent new-onset arrhythmic events until 30-day follow-up, while over half of early new-onset LBBB recovered within 30 days after discharge. Smartwatch ECG was safe and effective for home-based management of TAVR recipients. Large-scale prospective studies are needed to analyze the clinical and economic impact of smartwatch ECG, further prompting the establishment of recommendations for TAVR management at home.

## Data Availability

All data generated or analyzed during this study are included in this published article.

## Abbreviations

TAVR, transcatheter aortic valve replacement; AF, atrial fibrillation; AVB, atrial ventricular block; PPI, permanent pacemaker implantation; ECG, electrocardiogram; SCD, severe conduction disturbance; OR, odds ratio; CI, confidence intervals; PPV, positive predictive value; NPV, negative predictive value.

## Supplementary Material

Supplementary material associated with this article can be found, in the online version, at https://doi.org/10.31083/j.rcm2401011.
