# Supplementary material for: Ambulatory Smartwatch ECG Monitoring among Patients Undergoing Transcatheter Aortic Valve Replacement Early after Discharge: An Observational Study
Source: Rev Cardiovasc Med. 2023 Jan 4;24(1):11. doi: 10.31083/j.rcm2401011 (PMC11270444; doi:10.31083/j.rcm2401011)
Supplement: Supplementary file 1 [file 2153-8174-24-1-011-s1.zip › 2153-8174-24-1-011-s1.docx]

**Supplementary Appendix**

**Supplementary Figure 1.** Configuration of Huami smartwatch from Anhui Huami Information Technology Co. Ltd.


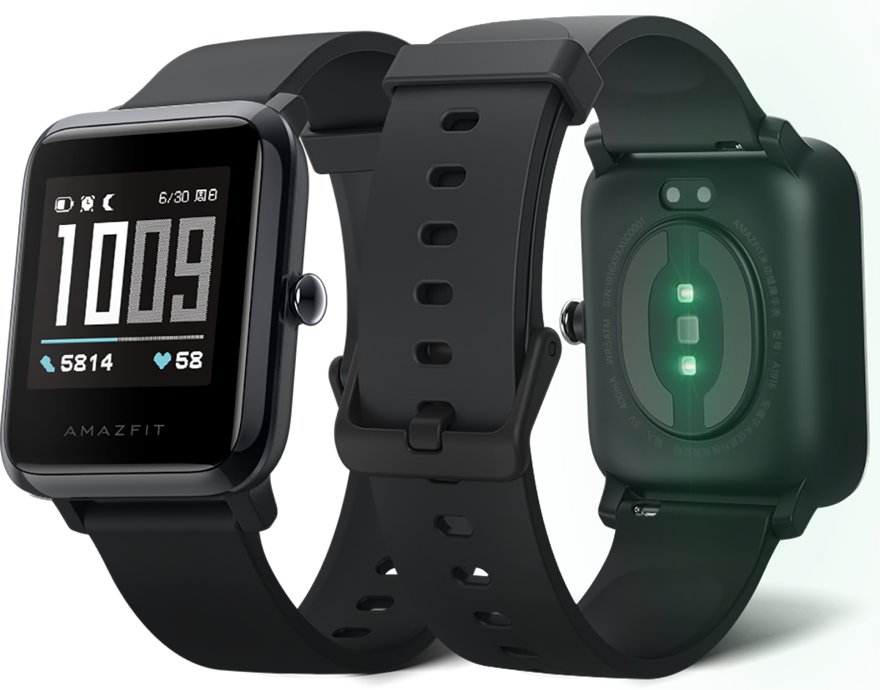


**Analysis of daily sleep, step and heartbeat for 30 days after discharge**

There were 67 patients with complete 24-hour heartbeat data (which meant smartwatch wearing time more than 22 hour per day) within 30 days after discharge were included in the analysis of variation of daily heartbeat, sleep and step counts from pre-operative stage to 4 weeks after discharge. The analysis of variation of 24-hour heartbeat excluded 9 patients (13.4%, 9/67) with persistent AF after discharge. After multiple pairwise comparison which corrected by false-positive rate error control, results indicated that 24-hour heartbeat, sleep duration, deep sleep duration, and step count did not differ significantly across time periods (Supplementary Figure 2). At pre-operative stage, post-operative stage, 1 week after discharge, 2 weeks after discharge, 3 weeks after discharge and 4 weeks after discharge, the mean 24-hour heartbeat were 110124.3 times, 108027.6 times, 107452.5 times, 109852.3 times, 109844.4 times and 107408.1 times, respectively; the mean 24-hour sleep duration were 410.0 minutes, 455.2 minutes, 454.0 minutes, 461.6 minutes, 426.1 minutes and 419.9 minutes, respectively; the mean 24-hour deep sleep duration were 101.8 minutes, 107.1 minutes, 103 minutes, 109.4 minutes, 101.2 minutes and 98.7 minutes, respectively; the mean 24-hour step counts were 3848.5 steps, 3736.3 steps, 3866.2 steps, 3202.1 steps, 3899.9 steps and 3840.9 steps, respectively.

Supplementary figure 2. Variation of 24-hour heartbeat (A), sleep duration (B), deep sleep duration (C) and step counts (D) from pre-operative stage to 4 weeks after discharge.


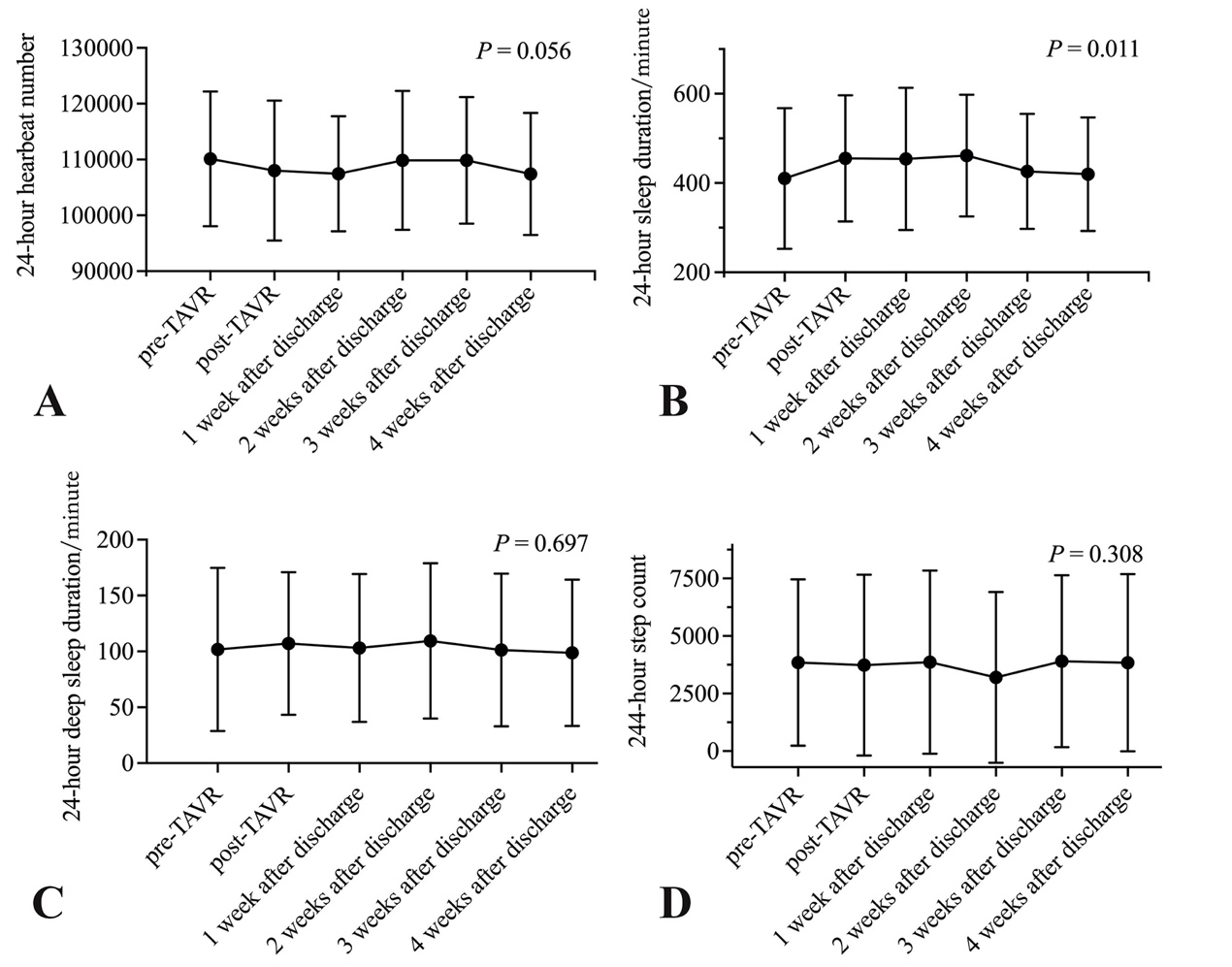


*Abbreviation: TAVR = transcatheter aortic valve replacement.

Supplementary Table 1. The diagnostic outcomes of AF, LBBB and RBBB from 24-hour Holter ECGs and smartwatch ECGs.

| Smartwatch ECG (N) | 24-hour Holter ECG (N) | | |
| --- | --- | --- | --- |
|  | Diagnosed | Undiagnosed | Total |
| AF | 10 | 0 | 10 |
| Non-AF | 3 | 83 | 86 |
| Unable diagnosis | 2 | 2 | 4 |
| Total | 15 | 85 | 100 |
| LBBB | 17 | 11 | 28 |
| Non-LBBB | 8 | 60 | 68 |
| Unable diagnosis | 1 | 3 | 4 |
| Total | 26 | 74 | 100 |
| RBBB | 3 | 2 | 5 |
| Non-RBBB | 3 | 88 | 91 |
| Unable diagnosis | 0 | 4 | 4 |
| Total | 6 | 94 | 100 |

ECG: electrocardiograph; AF: atrial fibrillation; LBBB: left bundle branch block; RBBB: right bundle branch block.

Supplementary Table 2. The diagnostic outcomes of AF, LBBB and RBBB from 12-lead ECGs and smartwatch ECGs.

| Smartwatch ECGs (N) | 12-lead ECGs (N) | | |
| --- | --- | --- | --- |
|  | Diagnosed | Undiagnosed | Total |
| AF | 46 | 6 | 52 |
| Non-AF | 1 | 365 | 366 |
| Unable diagnosis | 5 | 15 | 20 |
| Total | 52 | 386 | 438 |
| LBBB | 95 | 11 | 106 |
| Non-LBBB | 11 | 301 | 312 |
| Unable diagnosis | 8 | 12 | 20 |
| Total | 114 | 324 | 438 |
| RBBB | 19 | 4 | 23 |
| Non-RBBB | 23 | 372 | 395 |
| Unable diagnosis | 0 | 20 | 20 |
| Total | 42 | 396 | 438 |

AF: atrial fibrillation; LBBB: left bundle branch block; RBBB: right bundle branch block.

Supplementary Table 3. Regression analysis of predictors for delayed new-onset AF, delay new-onset SCD, overall new-onset AF, and overall new-onset permanent LBBB.

|  | Univariate regression  *OR* [95% *CI*] | *P* | Multivariate regression  *OR* [95% *CI*] | *P* |
| --- | --- | --- | --- | --- |
| Predictors for delayed new-onset AF |  |  |  |  |
| Age | 0.956 [0.873, 1.047] | 0.332 | 0.955 [0.852, 1.070] | 0.428 |
| Female | 1.813 [0.622, 5.284] | 0.275 | 3.565 [0.845, 15.044] | 0.084 |
| STS-PROM | 0.705 [0.429, 1.159] | 0.168 | 0.700 [0.323, 1.518] | 0.367 |
| Severe aortic stenosis | 0.343 [0.116 - 1.014] | 0.053 | 0.958 [0.192 – 4.785] | 0.958 |
| THV implantation depth | 1.107 [1.014 - 1.209] | 0.023 | 1.151 [1.000 - 1.324] | 0.050 |
| Predictors for delay new-onset SCD |  |  |  |  |
| Age | 1.001 [0.891 - 1.125] | 0.983 | 1.026 [0.884 - 1.191] | 0.736 |
| Male^†^ | 2.287 [0.518 - 10.089] | 0.275 | 2.084 [0.409 – 10.619] | 0.377 |
| STS-PROM | 0.954 [0.580 - 1.570] | 0.853 | 0.917 [0.491 - 1.712] | 0.785 |
| Prosthesis implantation depth | 0.929 [0.790, 1.091] | 0.367 | 0.938 [0.795, 1.107] | 0.448 |
| Early new-onset RBBB | 6.467 [1.032 - 40.533] | 0.046 | 6.512 [0.893 – 47.492] | 0.065 |
| Predictors for overall new-onset AF |  |  |  |  |
| Age | 1.004 [0.934, 1.080] | 0.908 | 1.019 [0.928, 1.118] | 0.694 |
| Female | 1.389 [0.560, 3.442] | 0.478 | 2.290 [0.736, 7.129] | 0.153 |
| STS-PROM | 0.930 [0.679, 1.275] | 0.654 | 0.857 [0.514, 1.429] | 0.555 |
| THV implantation depth | 1.116 [1.030 - 1.210] | 0.008 | 1.110 [0.991 - 1.243] | 0.072 |
| Severe aortic stenosis | 0.240 [0.093 - 0.621] | 0.003 | 0.630 [0.165 - 2.412] | 0.500 |
| Predictors for overall new-onset SCD |  |  |  |  |
| Age | 1.008 [0.939, 1.082] | 0.822 | 1.002 [0.907, 1.106] | 0.969 |
| Female | 1.716 [0.708, 4.160] | 0.232 | 1.618 [0.537, 4.871] | 0.392 |
| STS-PROM | 1.065 [0.809, 1.401] | 0.654 | 1.141 [0.698, 1.865] | 0.599 |
| THV implantation depth | 0.940 [0.854, 1.034] | 0.202 | 0.950 [0.856, 1.055] | 0.340 |
| baseline RBBB | 14.909 [1.585 - 140.224] | 0.018 | 21.659 [1.631, 287.551] | **0.020** |
| Pre-TAVR statins | 2.216 [0.906 - 5.418] | 0.081 | 3.793 [1.243, 11.574] | **0.019** |
| Predictors for overall new-onset permanent LBBB |  |  |  |  |
| Age | 1.013 [0.930, 1.102] | 0.769 | 1.005 [0.900, 1.123] | 0.924 |
| Female | 0.379 [0.114, 1.260] | 0.113 | 0.566 [0.133, 2.406] | 0.441 |
| STS-PROM | 1.316 [0.982 - 1.763] | 0.066 | 1.438 [0.981 – 2.106] | 0.063 |
| Renal dysfunction | 4.108 [0.876 - 19.259] | 0.073 | 1.979 [0.232 – 16.857] | 0.532 |
| Pre-TAVR PG_mean_ | 0.959 [0.921 - 0.999] | 0.044 | 0.960 [0.920 - 1.003] | 0.066 |

AF: atrial fibrillation; STS-PROM: Society of Thoracic Surgeons predicted rate of mortality; THV: transcatheter heart valve; SCD: severe conduction disturbances; RBBB: right bundle branch block; LBBB: left bundle branch block; TAVR: transcatheter aortic valve replacement; OR: odds ratio; CI: confidence interval.
